# Gastric cancer stem cells survive in stress environments via their autophagy system

**DOI:** 10.1038/s41598-021-00155-3

**Published:** 2021-10-19

**Authors:** Shingo Togano, Masakazu Yashiro, Go Masuda, Atsushi Sugimoto, Yuichiro Miki, Yurie Yamamoto, Tomohiro Sera, Shuhei Kushiyama, Sadaaki Nishimura, Kenji Kuroda, Tomohisa Okuno, Masaichi Ohira

**Affiliations:** 1 Department of Gastroenterological Surgery, Osaka City University Graduate School of Medicine, 1-4-3 Asahimachi, Abeno-ku, Osaka, 545-8585 Japan; 2 Molecular Oncology and Therapeutics, Osaka City University Graduate School of Medicine, Osaka, Japan; 3 Cancer Center for Translational Research, Osaka City University Graduate School of Medicine, Osaka, Japan

**Keywords:** Macroautophagy, Gastric cancer, Cancer stem cells

## Abstract

Cancer stem cells (CSCs) play an important role in the progression of carcinoma and have a high potential for survival in stress environments. However, the mechanisms of survival potential of CSCs have been unclear. The aim of this study was to clarify the significance of autophagy systems of CSCs under stress environments. Four gastric cancer cell line were used. Side population (SP) cells were sorted from the parent cells, as CSC rich cells. The expression of stem cell markers was examined by RT-PCR. The viability of cancer cells under starvation and hypoxia was evaluated. The expression level of the autophagy molecule LC3B-II was examined by western blot. The numbers of autophagosomes and autolysosomes were counted by electron microscope. SP cells of OCUM-12 showed a higher expression of stem cell markers and higher viability in starvation and hypoxia. Western blot and electron microscope examinations indicated that the autophagy was more induced in SP cells than in parent cells. The autophagy inhibitor significantly decreased the viability under the stress environments. These findings suggested that Cancer stem cells of gastric cancer might maintain their viability via the autophagy system. Autophagy inhibitors might be a promising therapeutic agent for gastric cancer.

## Introduction

It has been reported that cancer stem cells (CSCs), a unique subpopulation in tumors, play an important role for the progression of carcinomas^1–3^. CSCs has been known to be the prime sources of self-renewal, and to supply cancer cells^4–6^. CSCs have a high potential to survive various stresses such as starvation and hypoxia, resulting the increase of the proportion of CSCs^6,7^. However, the mechanisms responsible for the high survival capacity of CSCs under these stresses has remained to be unclear. The side population (SP) of cancer cells evaluated by flow cytometric analysis is known as a CSC-rich population. SP cells might be useful for the stemness of various carcinomas^8–10^.

Autophagy is an intracellular degradation and re-use system which is induced under various stresses such as starvation and hypoxia; it is a so-called “self-eating process” involving intracellular proteins, complexes, or organelles in the autophagosome^11–13^. The autophagosome is transported and fuses with the lysosome to generate the autolysosome, and the components are degraded by acidic hydrolases. The degradation products, including nucleotides, amino acids, fatty acids, and sugars are transported back and recycled into the general cell metabolism^11,14^. It has been reported that the autophagy systems involved in cancer might be associated with the survival of cancer cells^15^. However, the significance of the autophagy system of CSCs, especially under stress conditions, remains unclear. We then aimed to clarify the significance of the autophagy systems of CSCs in stress environments such as starvation and hypoxia in this study.

## Results

### SP fraction in 4 cell lines

**Figure 1:**
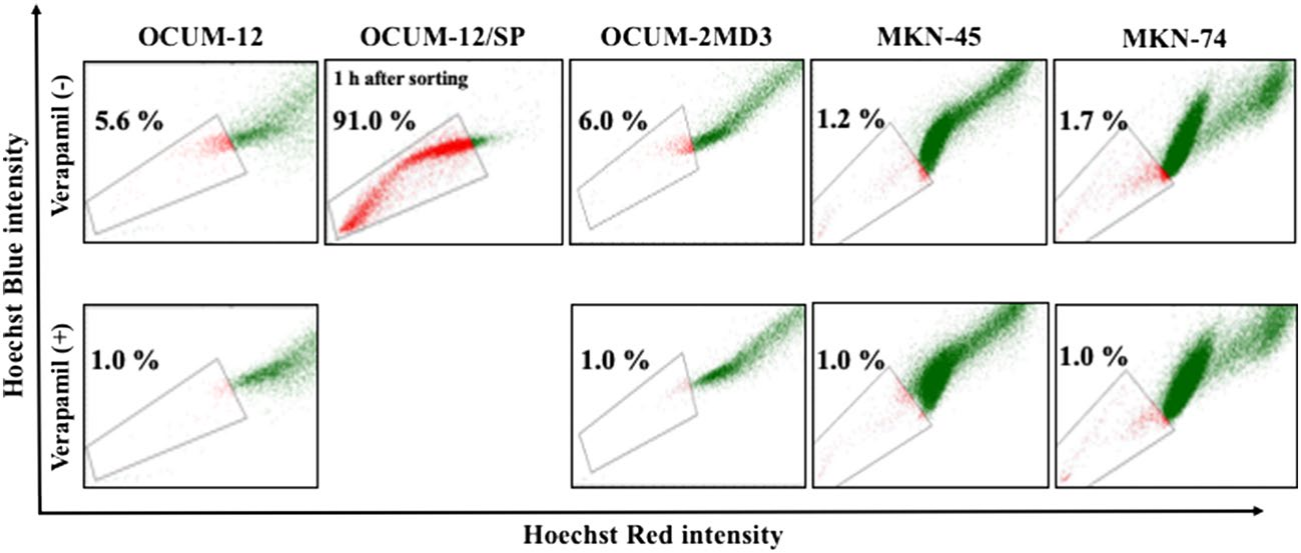
Representative picture of SP fraction. Cancer cells, which disappear in the presence of verapamil, are outlined and defined as the SP cells. The SP percentage of the parent OCUM-12, OCUM-2MD3, MKN-45 and MKN-74 cells was 5.6%, 6.0%, 1.2% and 1.7%, respectively. Since MKN-45 cells and MKN-74 cells have few SP fractions, MKN-45 and MKN-74 were excluded from the following examinations. To confirm the sorting accuracy, sorted SP cells of the parent OCUM-12 cells were re-analyzed 1 h later. The SP percentage of OCUM-12/SP was 91.0%.

SP cells were defined as the population that disappeared with the administration of verapamil (≤ 1%). Figure 1 shows representative pictures of the SP fraction determined by flow cytometric analysis. The SP percentage of the parent OCUM-12, OCUM-2MD3, MKN-45 and MKN-74 cells was 5.6%, 6.0%, 1.2% and 1.7%, respectively. The SP percentage of SP cells at 1 h after the sorting of the parent OCUM-12 cells (OCUM-12/SP cells) was 91.0%, which suggested that most of the cells sorted by flow cytometry were SP cells.

### Stem cell marker expression in parent cells and SP cells

**Figure 2:**
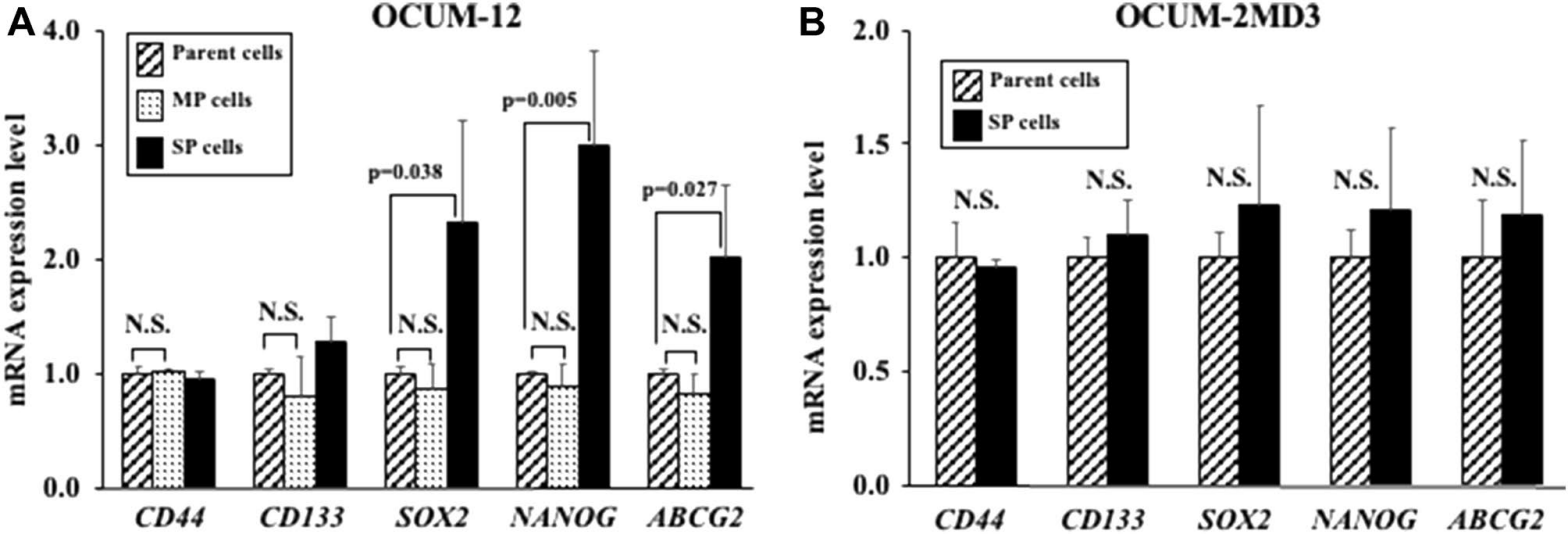
Expression of stem cell markers. (**A**) The mRNA expression levels of *SOX2*, *NANOG,* and *ABCG2* were significantly higher in the OCUM-12/SP cells (2.31-, 3.00-, and 2.02-fold; p = 0.038, 0.005, and 0.027, respectively) in comparison to the parent OCUM-12 cells. (**B**) No significant difference of stem cell markers was found between the parent OCUM-2MD3 cells and the OCUM-2MD3/SP cells.

The mRNA expression levels of *SOX2*, *NANOG*, and *ABCG2* were significantly higher in the OCUM-12/SP cells than in the parent OCUM-12 cells. On the other hand, no significant difference of stem cell markers was found between the parent OCUM-2MD3 cells and the OCUM-2MD3/SP cells (Fig. 2).

### Effect of starvation and hypoxic stress on the proliferation of OCUM-12 cells

**Figure 3:**
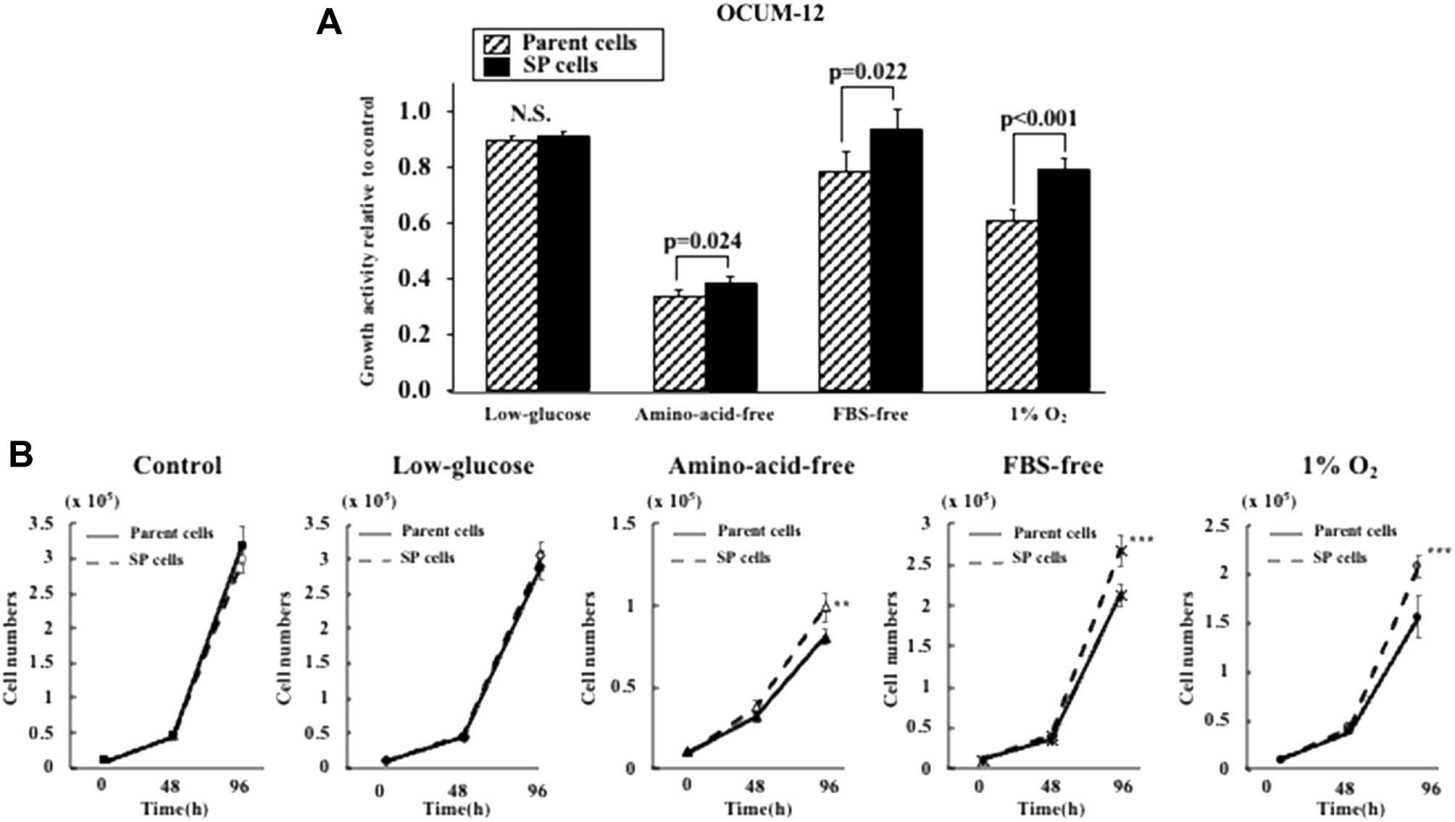
Proliferation activity of cancer cells under various stress environments. (**A**) Proliferation activity of cancer cells under starvation and hypoxic stress in MTT assay. The viability of SP cells was significantly high under amino-acid-free, FBS-free, and 1%O_2_ conditions (0.38 and 0.34; p = 0.024, 0.94 and 0.79; p = 0.022, 0.80 and 0.61; p < 0.001, relative to control; respectively), but was not significant under the low-glucose condition. (B) Growth curves of parent cells and SP cells. Cells seeded at a density of 1.0 × 10^4^/well in 12-well plates under each medium or hypoxia. The proliferative activity of the OCUM-12/SP cells was significantly higher than that of the parent OCUM-12 cells in amino-acid-free medium, FBS-free medium, and under a 1%O_2_ condition, but not in a low-glucose medium. *p < 0.05, **p < 0.01, *** p < 0.005.

We examined the effect of starvation and hypoxic stress on the proliferation activity of the parent OCUM-12 cells and the OCUM-12/SP cells. The proliferation of parent cells and SP cells was quantified using an MTT colorimetric assay and cell counting assay. Both assays showed the proliferative activity of the OCUM-12/SP cells was significantly higher than that of the parent OCUM-12 cells in amino-acid-free medium, FBS-free medium, and under a 1%O_2_ condition, but not in a low-glucose medium (Fig. 3).

### Effect of starvation and hypoxic stress on the SP fraction of OCUM-12 cells

**Figure 4:**
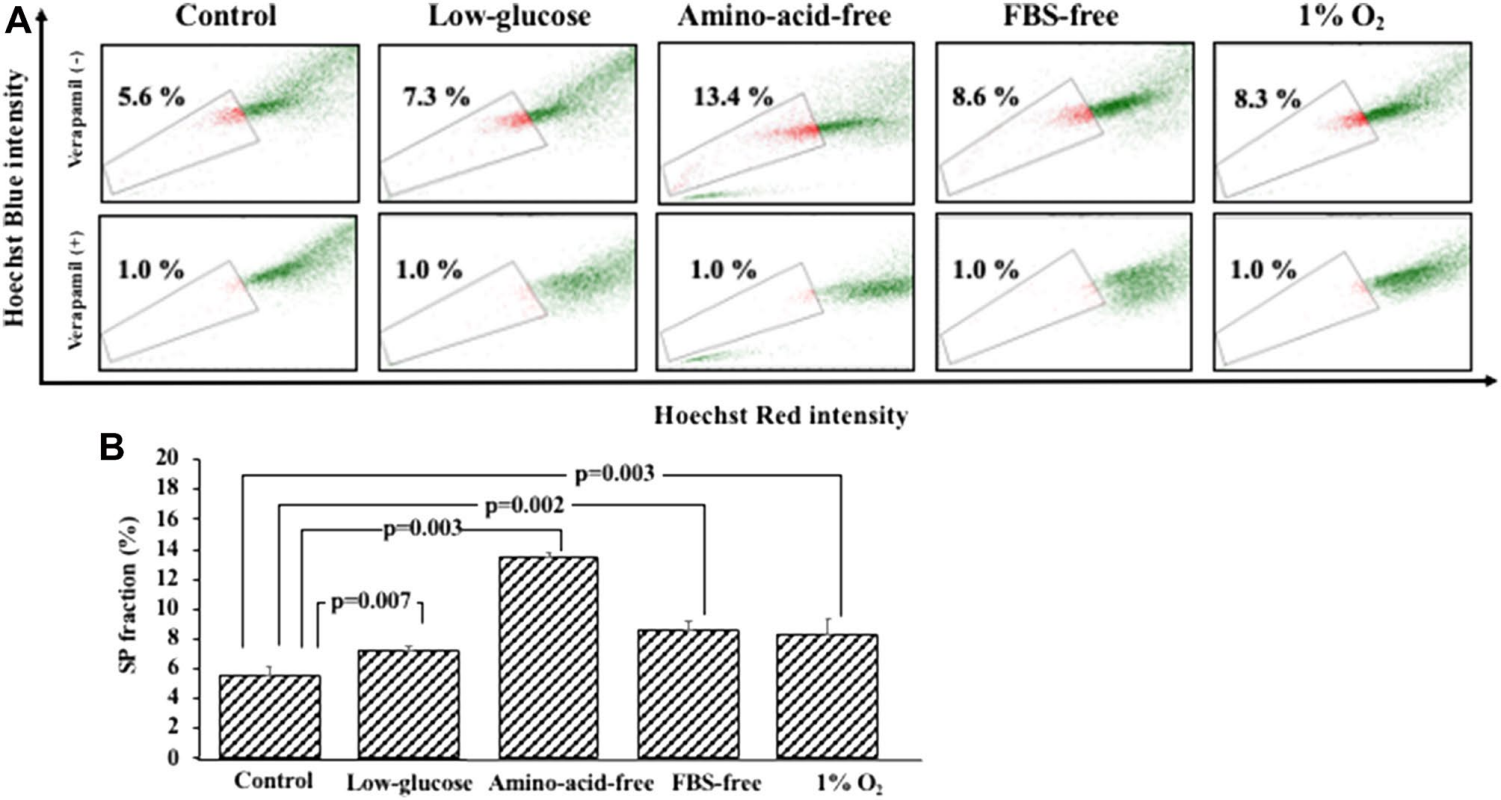
The SP fraction under starvation and hypoxic stress. (**A**) Representative picture of SP fraction under starvation and hypoxic stress. (**B**) The SP fraction was significantly high under starvation stress and hypoxic stress in comparison to the parent cells. The SP fractions under the low-glucose-medium, amino-acid-free medium, FBS-free medium, and 1%O_2_ conditions were 7.3 ± 0.2% (p = 0.007), 13.2 ± 0.4% (p = 0.003), 7.9 ± 0.7% (p = 0.002), and 7.7 ± 1.0% (p = 0.003) in comparison to the parent cells, erspectively.

The effect of starvation and hypoxic stress on the SP fraction was analyzed by flow cytometry. Starvation stress and hypoxia significantly increased the SP fraction (Fig. 4). The SP fraction observed when using the low-glucose medium, amino-acid-free medium, FBS-free medium, and 1%O_2_ condition was significantly higher than that of the control.

### The expression of autophagy marker of OCUM-12 cells in the mouse xenograft model

**Figure 5:**
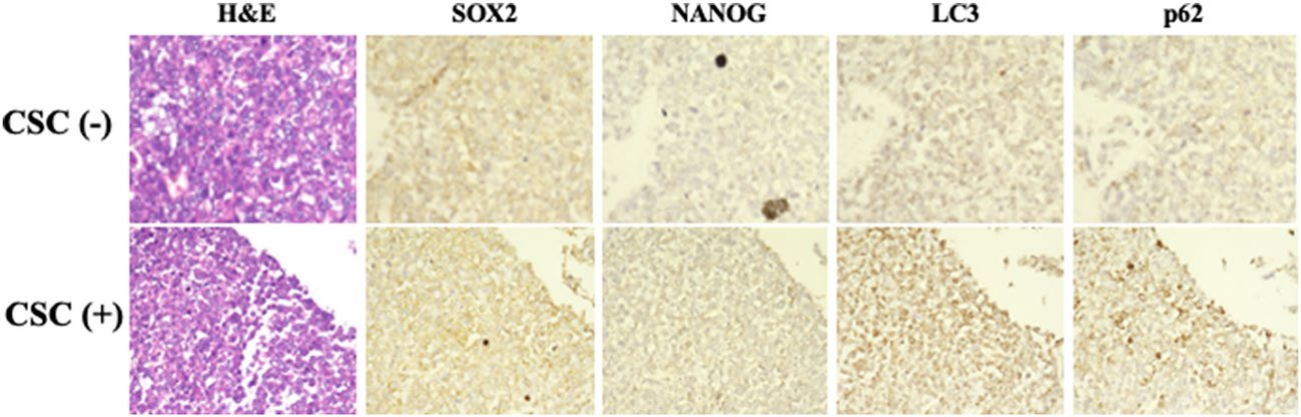
Immunohistochemical staining of xenografted tumor. The xenografted tumor by OCUM-12 cells were stained by cancer stem cells markers, SOX2 and NANOG. LC3B and p62 are autophagy markers. Original magnification: 100.

SOX2 and NANOG staining was performed to determine the CSCs of OCUM-12. LC3B and p62 staining was performed to evaluate the autophagic activity. Both positive of SOX2 and NANOG was evaluated as CSC-positive, and both positive of LC3B and p62 was evaluated as autophagy-positive. CSC-positive was significantly (p = 0.016) associated with autophagy-positive (Fig. 5).

### Effect of starvation and hypoxic stress on autophagy

**Figure 6:**
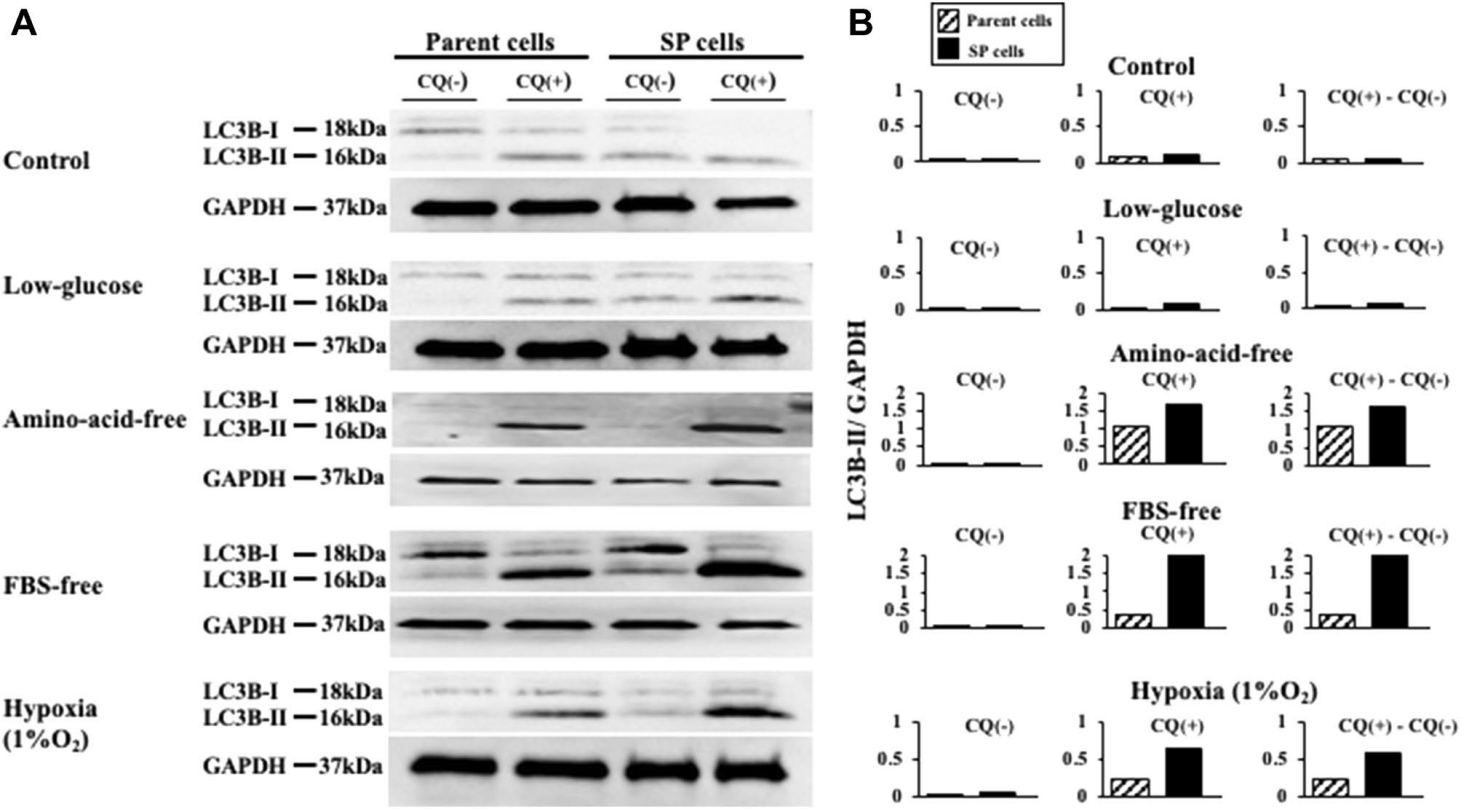
Autophagy flux of OCUM-12 cells. (**A**) Autophagy flux assay. (**B**) Autophagic flux was determined by subtracting the band intensity of the LC3B-II western blot in the presence of chloroquine (CQ +) and the corresponding treatment in the absence of chloroquine (CQ −). The expression of LC3B-II was higher in the OCUM-12/SP cells than in the parent OCUM-12 cells under amino-acid-free, FBS-free, and 1%O_2_ conditions (LC3B/GAPDH ratio (CQ +)—LC3B/GAPDH ratio (CQ −); 1.61 and 1.07, 2.19 and 0.35, 0.58 and 0.22, respectively). The difference in LC3B-II expression was greatest under the FBS-free medium. The LC3B-II expressions under the control medium and low-glucose medium were slight (0.04 and 0.06, 0.07 and 0.02, respectively). The displayed membrane were cut prior to hybridization. The full-length original, un-processed blot performed with each antibody, LC3B and GAPDH, which confirms specific detection of the target antigen was presented in Supplement Figs. S1 and S2.

**Figure 7:**
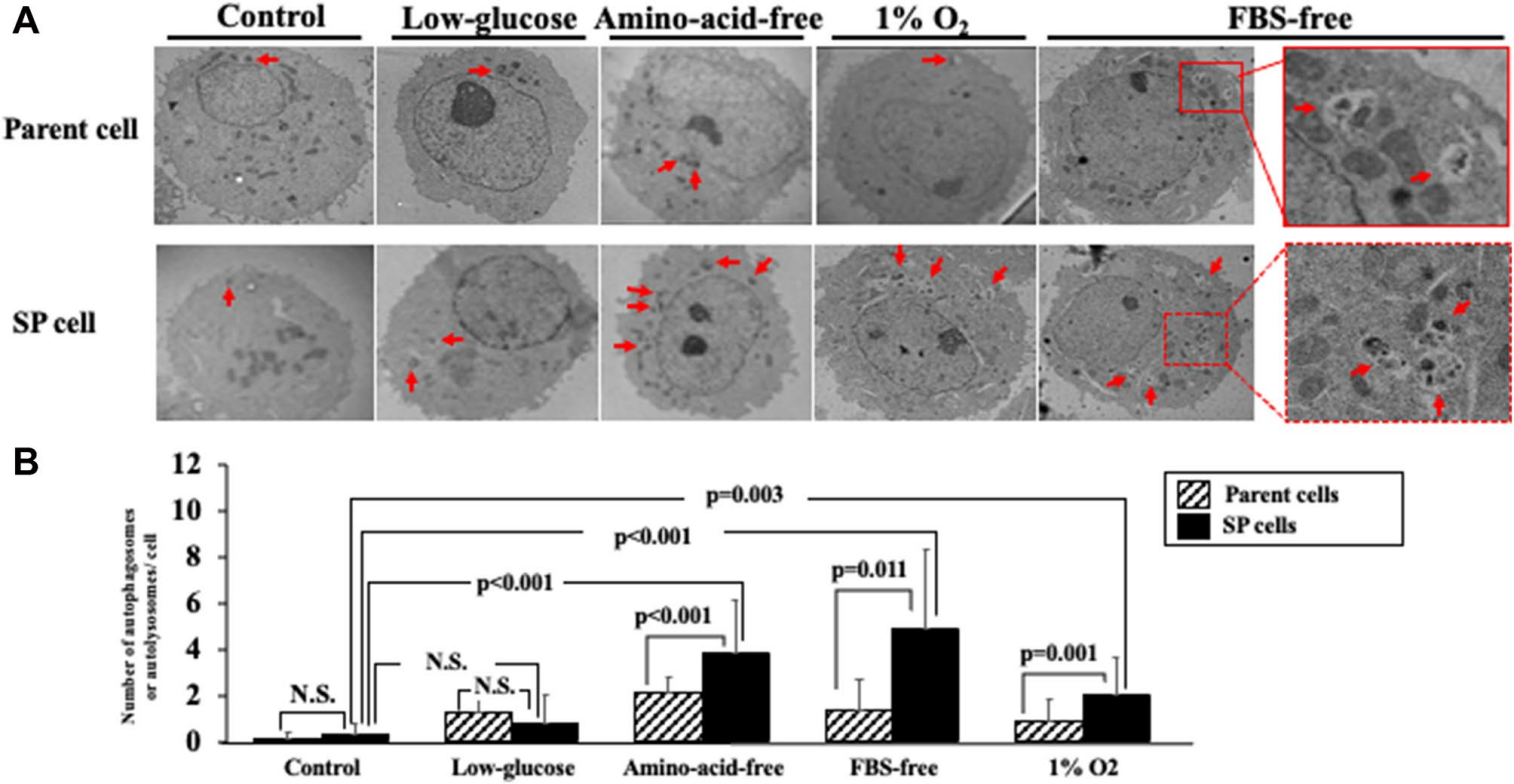
Molecular structure of autophagy in OCUM-12 cells*.* (**A**) Electron microscopic pictures of autophagosomes and autolysosomes. Arrows, autolysosomes or autophagosomes. (**B**) There was no significant difference between the OCUM-12/SP cells and the parent OCUM-12 cells in the control medium and low-glucose medium, but a significant increase in the number of autophagosomes and autolysosomes in the OCUM-12/ SP cells was observed under the Amino-acid-free medium (p < 0.001), FBS-free medium (p = 0.011), 1% O_2_ (p = 0.001).

The LC3B-II expression in the parent OCUM-12 cells was greatest when using the amino-acid-free medium, while that in the OCUM-12/SP cells was greatest with the FBS-free medium. With the control medium and low-glucose medium, the LC3B-II expression was slight in both types of cells. The LC3B-II expression when using the amino-acid-free medium, FBS-free medium, and 1%O_2_ condition was higher in the OCUM-12/SP cells than in the parent OCUM-12 cells, and the difference in LC3B-II expression was greatest with the FBS-free medium (Fig. 6). In addition, the numbers of autophagosomes and autolysosomes were counted using an electron microscope (Fig. 7A). A significant increase in the numbers of autophagosomes and autolysosomes in OCUM-12/SP cells was observed when using the amino-acid-free medium (p < 0.001), FBS-free medium (p = 0.011), 1%O_2_ condition (p = 0.001), while no significant difference was found in the control medium and low-glucose medium (Fig. 7B).

### The effect of an autophagy inhibitor, CQ, on the proliferation of OCUM-12 cells

**Figure 8:**
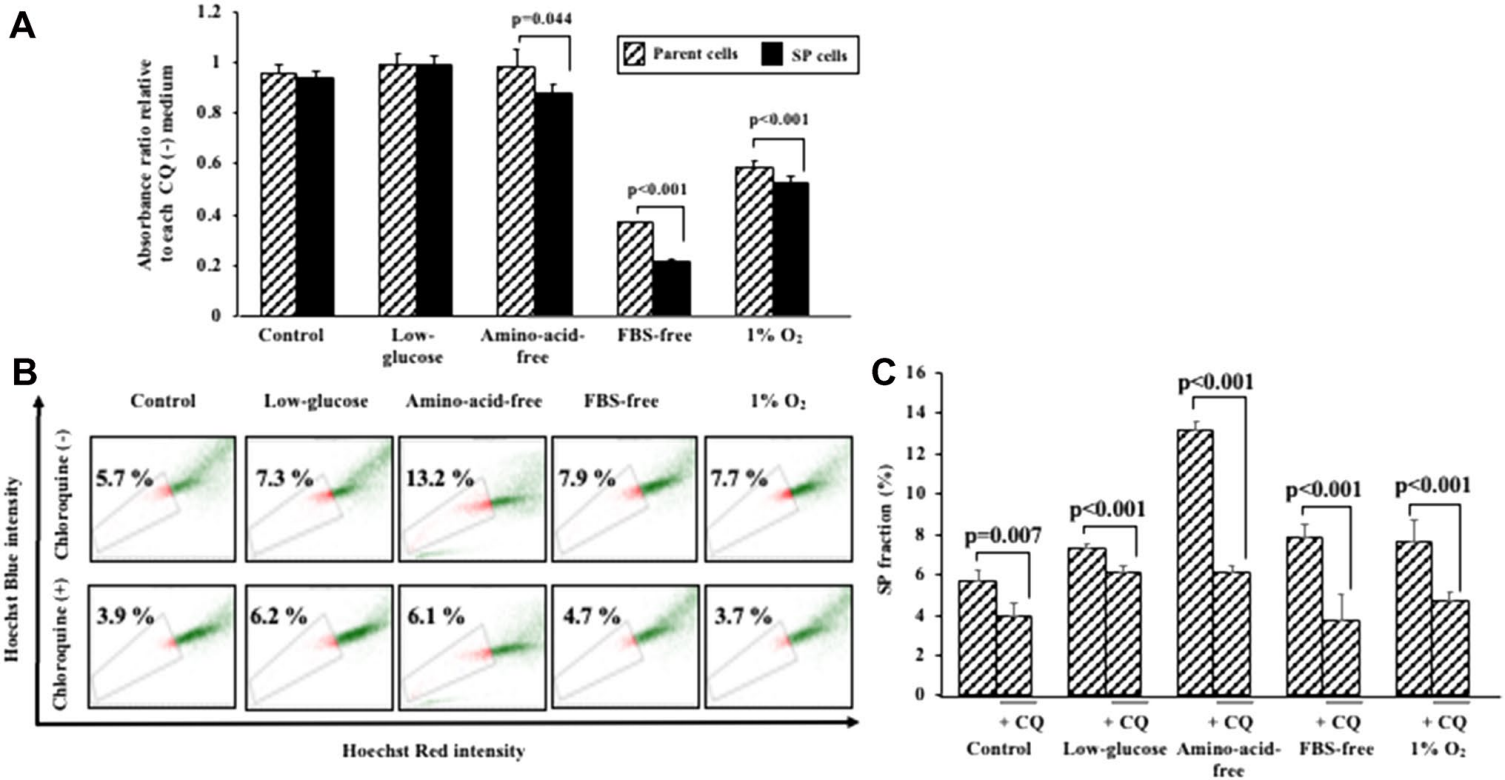
The cytotoxicity of the autophagy inhibitor on OCUM-12 cells. (**A**) The cytotoxicity of the autophagy inhibitor on the parent OCUM-12 cells and the OCUM-12/SP cells. Chloroquine (CQ) significantly decreased the viability of the OCUM-12/SP cells under amino-acid-free medium, FBS-free medium, and 1%O_2_ conditions (absorbance ratio relative to each CQ(−) medium; 0.88 and 0.99; p = 0.044, 0.21 and 0.37; p < 0.001, 0.53 and 0.59; p < 0.001, respectively). (**B**) Effect of the autophagy inhibitor on the SP fraction of OCUM-12 under starvation and hypoxic stress. The SP fraction was decreased under all conditions including in the control medium following the addition of CQ. (**C**) The SP fraction in the OCUM-12 cells was significantly reduced with CQ under amino-acid-free medium (13.2% to 6.1%), FBS-free medium (7.9% to 3.7%), and 1%O_2_ conditions (7.7% to 4.7%) compared to the control medium.

The proliferation activity of OCUM-12/SP cells in the amino-acid-free medium, FBS-free medium, and under the 1%O_2_ condition was significantly decreased following the addition of the autophagy inhibitor, CQ, in comparison to the parent OCUM-12 cells (Fig. 8A).

### The effect of CQ on the proportion of SP cells

CQ reduced the SP fraction. CQ combined with amino-acid-free medium, FBS-free medium, and under the 1%O_2_ condition greatly reduced the SP fraction compared to the control medium (Fig. 8B,C).

## Discussion

We aimed to clarify the significance of the autophagy systems of CSCs under stress environments such as starvation and hypoxia. It has been reported that SP cells of gastric cancer possess cancer stem-like properties^8,9^. We then used the SP cells of OCUM-12, OCUM-2MD3, MKN-45 and MKN-74 cells as CSCs in this study. Since MKN-45 cells and MKN-74 cells have few SP fractions, MKN-45 and MKN-74 were excluded from the following examinations. The SP percentage of OCUM-12/SP cells sorted by flow cytometry was 91.0%, while that of the parent OCUM-12 cells was 5.6%, which suggested that flow cytometry could successively select SP cells. Next, we evaluated the stemness of the OCUM-12/SP cells and the OCUM-2MD3/SP cells by RT-PCR using stem cell markers. The OCUM-12/SP cells showed higher expression of *SOX2*, *NANOG* and *ABCG2*, in comparison to the parent OCUM-12 cells. In contrast, no significant difference of stem cell markers was found between the parent OCUM-2MD3 cells and the OCUM-2MD3/SP cells. Then, OCUM-2MD3 was unfortunately excluded from the following study because the evidence of stemness of OCUM-2MD3/SP cells is necessary for the following examinations. The OCUM-12/SP cells could survive under stress conditions such as free amino acid, free FBS, or hypoxia in comparison to the parent OCUM-12 cells. These findings suggested that the OCUM12/SP cells possess cancer stem-like properties, as previously reported^8^.

Autophagic activity of OCUM-12 cells in the mouse xenograft model was measured using LC3B and p62 antibodies in immunohistochemical staining. CSC-positive, both positive of SOX2 and NANOG, was significantly associated with autophagy-positive, both positive of LC3B and p62. The interpretation of p62 positivity in immunohistochemical staining remains to be controversial. It has reported that the accumulation of p62 may indicate the inhibition of autophagy^16^. On the other hand, it has also reported that the accumulation of p62 may indicate autophagy-induced autophagosomes^17,18^. In this study, we determined that the both positive expression of LC3B and p62 of the xenografted tumor by OCUM-12 cells may indicate as the autophagy positive, because the CSC-rich OCUM-12/SP cells significantly increased the number of autophagosomes in compared to the parent OCUM-12 cells by electron microscopic examination.

LC3B-I is conjugated and forms LC3B-II, which is recruited to autophagosomal membranes. Autophagosomes fuse with lysosomes to form autolysosomes and are degraded by lysosomal hydrolases. The autophagosomal marker LC3B-II reflects starvation-induced autophagic activity^19^. Western blot of LC3B-II indicated that the autophagy system was induced in both the parent cells and SP cells in FBS starvation, amino acid starvation, and hypoxia (1%O_2_ condition), but that of SP cells was greater than that of parent cells. The number of autophagosomes and autolysosomes observed by electron microscopy was also increased in SP cells under FBS starvation, amino acid starvation and hypoxia (1%O_2_ condition), which supported the results of Western blot. The growth activity of SP cells was greater than that of parent cells under these stress environments. These findings might suggest that the survival potential of CSCs was greater than parent cells inducing autophagy system.

The autophagy inhibitor, CQ, significantly decreased the growth activity of the OCUM-12/SP cells under FBS starvation, amino acid starvation, and hypoxia (1%O_2_ condition), in compared to the parent OCUM-12 cells. The heterogenicity of starvation and hypoxic lesions in the tumor microenvironment is often associated with malignant transformation of solid tumors^20^. CSCs might survive under these stress environments via autophagy systems, and the CQ might decrease the survival activity of the CSCs of gastric cancer under stress environments. There are only two reports about the effect of CQ on CSCs in ovarian cancer^21^ and hepatocellular carcinoma^22^. This is the first report of the inhibitory effect of the autophagy inhibitor, CQ, on the proliferation of gastrointestinal CSCs. Gastrointestinal CSCs have been proposed to play an important role in the progression of carcinomas including the metastasis and chemoresistance of cancer cells. These findings suggested that the autophagy inhibitor might be useful for advanced stage patients with metastatic carcinoma or chemo-resistant gastric carcinoma.

In conclusion, CSCs of gastric cancer might maintain their viability under the stress environments of starvation and hypoxia via the autophagy system. Autophagy inhibitors might be promising therapeutic agents for gastric cancer.

## Methods

### Cell line

Four human gastric cancer cell line, OCUM-12 (RRID: CVCL_8380), OCUM-2MD3 (RRID: CVCL_8385), MKN-45 (RRID: CVCL_0434) and MKN-74 (RRID: CVCL_2791) were used as parent cells. OCUM-12^20^ and OCUM-2MD3^23^ were established at our laboratory. MKN-45 and MKN-74 were provided from JCRB Cell Bank^24^ (Osaka, Japan). All cell lines in this study were authenticated by STR profiling before distribution.

### Cell culture

Cells were cultured with each medium or condition: Dulbecco’s modified Eagle medium (DMEM) with high glucose 4500 mg/L and 16 amino acids (WAKO, Osaka, Japan) and 10% Fetal bovine serum (FBS; SIGMA, St. Louis, MO) as control medium, DMEM with low glucose 1000 mg/L and 16 amino acids and 10% FBS as low-glucose medium, DMEM with high glucose 4500 mg/L and no amino acids (WAKO) and 10% FBS as amino-acid-free medium, DMEM with high glucose 4500 mg/L and 16 amino acids, without FBS as FBS-free medium. These media were also contained penicillin and streptomycin, and 0.5 mM sodium pyruvate, and were incubated at 37 °C under 21% oxygen condition. Another condition was hypoxia: the control medium under 1%O_2_. This study was approved by the Osaka City University ethics committee (approval number 924).

### SP analysis using flow cytometry

SP cells were defined as the subset of cells that exhibited a low Hoechst33342 (SIGMA) staining pattern and disappeared with use of verapamil (SIGMA), as previously reported^8,10,25^. The cancer cells were suspended at 5 × 10^5^ cells/mL in DMEM. These cells were then incubated at 37 °C with agitation in water bath for 60 min with 5 μg/mL Hoechst 33342, either alone or in the presence of 50 μg/mL verapamil. After incubation, 1 μg/mL Propidium iodide (Becton Dickinson, Franklin Lakes, NJ, USA) was added and then filtered through a 40 μg cell strainer (Becton Dickinson) to obtain single-suspension cells. Analyses and sorting were performed using BD FACSAria II (Becton Dickinson). Hoechst red and blue are optical filters for measuring fluorescence emission. Hoechst 33342 was excited with the UV laser at 350 nm, and fluorescence emission was measured with 405 = BP30 (Hoechst blue) and 570 = BP20 (Hoechst red) optical filters. The SP cells, which disappear in the presence of verapamil, are outlined and shown as a percentage of the total cell population.

### Quantitative real-time reverse transcription-polymerase chain reaction (RT-PCR)

RT-PCR was performed as follows. A total of 1 × 10^5^ number of SP cells were sorted by flow cytometry. The total cellular RNA of parent cells and SP cells was extracted using Trizol reagent (Invitrogen, Carlsbad, CA, USA) and Direct-zol™ RNA MiniPrep (Zymo Research, Irvine, California, USA). cDNA was prepared from 1000 μg RNA using ReverTra Ace qPCR RT Master Mix (TOYOBO, Osaka, JAPAN). Quantitative real-time PCR was performed using the THUNDERBIRD SYBR qPCR MIX (TOYOBO). Aliquots of cDNA were amplified for 40 cycles consisting of 15 s of denaturing at 95 °C, 60 s of annealing and extension at 60 °C. Each PCR was performed in triplicate. The expression of each human gene was then normalized using ACTβ expression as an internal control.

To determine fold changes in each gene, RT-PCR was performed on the ABI Prism 7500 (Applied Biosystems, Foster City, CA, USA), with commercially available gene expression assays (Applied Biosystems) for, CD44, CD133, SOX2, NANOG, and ATP-binding cassette, sub-family G, member 2 (ABCG2). ACTβ was used as an internal standard to normalize mRNA levels. The primer sequences were listed in Supplementary Table 1.

### Effect of starvation or hypoxia and chloroquine on SP fraction

Each cell was incubated 12 h in each medium or hypoxia, either alone or in the presence of 10 μM chloroquine (CQ; SIGMA). Cells were suspended at 5 × 10^5^ cells/ mL in PBS. SP fraction was analyzed by flow cytometry.

### Cell growth assay and cytotoxic assay

The proliferation of parent cells and SP cells and cytotoxicity of CQ were quantified using an MTT colorimetric assay (Dojindo, Kumamoto, Japan) and cell counting assay. Each cell was seeded at a cell density of 1.5 × 10^3^/well in 96-well plates for MTT colorimetric assay, and seeded at a cell density of 1.0 × 10^4^/well in 12-well plates for cell counting assay in each medium and hypoxia, and incubated 96 h at 37 °C for proliferation assay. For cytotoxicity assay, each cell was seeded at a cell density of 3.0 × 10^3^/well in 96-well plates in each medium and hypoxia with or without 10 μM CQ, and incubated 48 h at 37 °C. MTT solution was added to each well at 500 μg/ ml. The cells were incubated for 2 h and lysed in dimethyl sulfoxide and the absorbance value was analyzed using a Varioskan LUX (Thermo Fisher scientific, Waltham, MA, USA) at a wavelength of 535 nm.

### Immunohistochemical techniques

Hematoxyline-eosin staining and the immunohistochemical determination of SOX2, NANOG, LC3B and p62 were examined using the subcutaneous xenografted tumors. The specimens were incubated with SOX2 (sc-398254, Santa Cruz, Dallas, TX. 1:50), NANOG (sc-374001, Santa Cruz, Dallas, TX. 1:50), LC3B (sc-271625, Santa Cruz, Dallas, TX. 1:50) and p62 (sc-28359, Santa Cruz, Dallas, TX. 1:50) overnight at 4 ˚C. The slides were treated with streptavidin-peroxidase reagent, and were incubated in PBS diaminobenzidine and 1% hydrogen peroxide v = v, followed by counterstaining with Mayer’s hematoxylin. Ten randomly fields were evaluated.

### Western blot analysis

Each cell was seeded at a cell density of 5.0 × 10^4^/well in 6-well plates under each medium or hypoxia in absence (− CQ) or presence (+ CQ) of 10 μM CQ and incubated 6 h at 37 °C. Cell lysates were made by standard methods. The protein concentration of each sample was measured using a Bio-Rad protein assay kit II (Bio-Rad Laboratory, Richmond, CA, USA). For SDS-PAGE, 2.5 μg of proteins from each sample were subjected to electrophoresis on 10–15% polyacrylamide gels. Proteins were electrophoretically transferred to polyvinylidene difluoride membranes with a tank transfer systems (Bio-Rad Laboratory), then blocked with buffer containing 3.0% skim-milk and 0.1% Tween-20 in Tris-buffered saline (TBST) at room temperature for 1 h. The blotting membranes were cut prior to hybridization with primary antibodies. Primary antibody of a microtubule-associated protein-light chain 3B (LC3B; L7543, SIGMA) was used at 1:1000 dilution, GAPDH (sc-47724, Santa Cruz, Dallas, TX) was used at 1:5000 dilution in TBST containing 3.0% skim-milk. The membranes were incubated with primary antibody overnight at 4 °C, washed (3 × 5 min) with TBST, followed by incubation with a horseradish peroxidase-conjugated secondary antibodies (NA931V and NA934V, GE Healthcare, Chicago, IL, USA) in TBST for 1 h at room temperature, then washed (3 × 10 min) with washing buffer. Detection of chemiluminescence was performed with immunoStar LD (WAKO) following the manufacturer's instructions. Band intensity were estimated using ImageQuant TL (GE).

### Counting the autophagy flux by electron microscope

Each cell was incubated 12 h in each medium and hypoxia, and was trypsinized and pelleted. The cells were then suspended and fixed 30 min at 4 °C in 2.5% glutaraldehyde with 2% paraformaldehyde in 0.1% M Phosphate buffer. The cells were then rinsed 4 times in 0.1% M Phosphate buffer and spun down into 3% agarose at 55 °C, and cooled to form blocks. The agarose blocks were incubated in 1% osmium tetroxide in 0.1 MPB for 2 h at room temperature. The agarose blocks were rinsed 4 times in 0.1% MPB and dehydrated in graded steps of alcohol and embedded in propylene oxide. Following polymerization overnight at 60 °C, 80-nm sections were cut on a UltracutUCT (Leica, Wien, Austria) and picked up on copper grids. The grids were post-stained in uranyl acetate and Reynolds solution. The sections were observed in a Talos F200C (FEI, Hillsboro, OR, USA). The numbers of autophagosomes and autolysosomes in 10 cells was counted under each condition.

### Statistical analysis

The statistical analysis was done using the Student's t-test. Statistical significance was set at ≤ 0.05.

## Supplementary Information

Supplementary Table 1.

Supplementary Figure 1.

Supplementary Figure 2.
