# Supplementary material for: Gastric cancer stem cells survive in stress environments via their autophagy system
Source: Sci Rep. 2021 Oct 19;11:20664. doi: 10.1038/s41598-021-00155-3 (PMC8526688; doi:10.1038/s41598-021-00155-3)
Supplement: Supplementary file 1 — Supplementary Table 1. [file 41598_2021_155_MOESM1_ESM.docx]

**Gastric Cancer Stem Cells Survive in** **Stress Environments via Their Autophagy System**

Shingo Togano^1,2^, Masakazu Yashiro^1,2,3,^ *, Go Masuda^1,2^, Atsushi Sugimoto^1,2^, Yuichiro Miki^1,2^, Yurie Yamamoto^2,3^, Tomohiro Sera^1,2^, Shuhei Kushiyama^1,2^, Sadaaki Nishimura^1,2^, Kenji Kuroda^1,2^, Tomohisa Okuno^1,2^, and Masaichi Ohira^1^

^1^ Department of Gastroenterological Surgery, Osaka City University Graduate School of Medicine, Osaka, Japan.

^2^ Molecular Oncology and Therapeutics, Osaka City University Graduate School of Medicine, Osaka, Japan.

^3,^ Cancer Center for Translational Research, Osaka City University Graduate School of Medicine, Osaka, Japan.

**Supplementary Table 1.** The primer sequences.

ACTβ

Forward: 5’-CCTCGCCTTTGCCGATCCG -3’

Reverse: 5’-TTGCACATGCCGGAGCCG-3’

CD44

Forward: 5’-AGCCTGGCGCAGATCGATT-3’

Reverse: 5’- TCCGTCCGAGAGATGCTGTAG -3’

CD133

Forward: 5’-CACTGAGCACTCTATACCAAAGC -3’

Reverse: 5’- TGAGCAAAATCCAGAGAAGCTAG -3’

SOX2

Forward: 5’-TGCAGTACAACTCCATGACCAG -3’

Reverse: 5’- GCGAGTAGGACATGCTGTAGG -3’

NANOG

Forward: 5’-GCCTGAAGAAAACTATCCATCCT -3’

Reverse: 5’- GCTGTCCTGAATAAGCAGATCCA -3’

ABCG2

Forward: 5’-GGGTTCTCTTCTTCCTGACGACC -3’

Reverse: 5’- TGGTTGTGAGATTGACCAACAGACC -3’
