# Supplementary figures and images for: Gastric cancer stem cells survive in stress environments via their autophagy system
Source: Sci Rep. 2021 Oct 19;11:20664. doi: 10.1038/s41598-021-00155-3 (PMC8526688; doi:10.1038/s41598-021-00155-3)

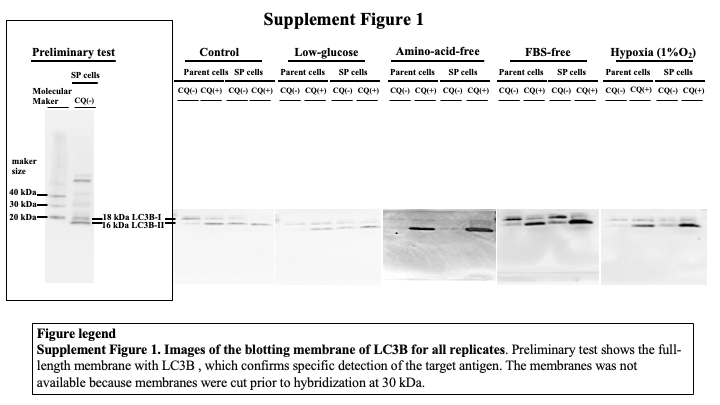

Supplement: Supplementary file 2 — Supplementary Figure 1. [file 41598_2021_155_MOESM2_ESM.tiff]

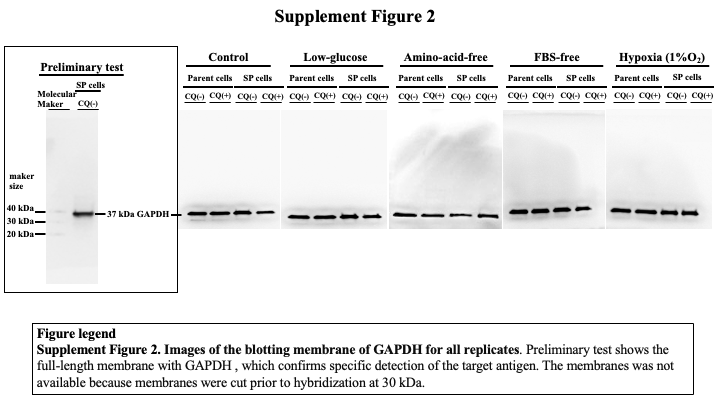

Supplement: Supplementary file 3 — Supplementary Figure 2. [file 41598_2021_155_MOESM3_ESM.tiff]
